# Thermal stress, p53 structures and learning from elephants

**DOI:** 10.1038/s41420-024-02109-w

**Published:** 2024-08-07

**Authors:** Konstantinos Karakostis, Monikaben Padariya, Aikaterini Thermou, Robin Fåhraeus, Umesh Kalathiya, Fritz Vollrath

**Affiliations:** 1 Institut de Biotecnologia i de Biomedicina, Universitat Autònoma de Barcelona, Bellaterra, Barcelona, Spain; 2 Inserm UMRS1131, Institut de Génétique Moléculaire, Université Paris 7, Hôpital St. Louis, Paris, France; 3 International Centre for Cancer Vaccine Science, University of Gdansk, ul. Kładki 24, Gdansk, Poland; 4 Research Centre for Applied Molecular Oncology (RECAMO), Masaryk Memorial Cancer Institute, Brno, Czech Republic; 5 Department of Medical Biosciences, Umeå University, Umeå, Sweden; 6 Department of Biology, University of Oxford, Oxford, UK; 7 Save the Elephants Marula Manor, Karen, P.O. Box 54667, Nairobi, Kenya

**Keywords:** Apoptosis, Stress signalling, Tumour-suppressor proteins, Molecular modelling

## Abstract

As species adapt to climatic changes, temperature-dependent functions of p53 in development, metabolism and cancer will adapt as well. Structural analyses of p53 epitopes interacting in response to environmental stressors, such as heat, may uncover physiologically relevant functions of p53 in cell regulation and genomic adaptations. Here we explore the multiple p53 elephant paradigm with an experimentally validated in silico model showing that under heat stress some p53 copies escape negative regulation by the MDM2 E3 ubiquitin ligase. Multiple p53 isoforms have evolved naturally in the elephant thus presenting a unique experimental system to study the scope of p53 functions and the contribution of environmental stressors to DNA damage. We assert that fundamental insights derived from studies of a historically heat-challenged mammal will provide important insights directly relevant to human biology in the light of climate change when ‘heat’ may introduce novel challenges to our bodies and health.

## Adaptation of the p53 machinery to environmental stressors

Adaptation to environmental changes drives species survival. Factors including temperature fluctuations or hypoxic environments influence genome regulation and lead to convergent evolutionary adaptations of p53 [1, 2]. Temperature changes affect cellular mechanisms such as metabolism, protein synthesis and cell cycling [3]. The growth of tumours induces increased cold stress in tumour-bearing mice [4]; and human clinical tests suggest that killer-cell cytotoxicity can be used against tumour target cells using thermal stress in the range of fever temperatures [5–7]. Recent studies on single cells revealed that the levels of the p53 transcription factor, widely known for its function as tumour suppressor controlling cell cycle, are dynamically modulated by temperature changes and mild hyperthermia inducing p53 response in the absence of genotoxic stress [3]. From an evolutionary viewpoint, the thermodynamic stability (melting temperature) of p53 in homeotherm vertebrates is correlated with body temperature [8].

A proposed mechanism linking p53 and thermoregulation considers that p53 plays multiple roles in the metabolism of adipose tissue, leading to the control of thermoregulation, energy metabolism and homeostasis [9]. Inhibition of p53 transcriptional activity decreases the expression of several thermogenic markers in adipocytes affecting body energy homeostasis [9], and the role of p53 is well established concerning the immune landscape of tissue cells [10] and the lipid metabolism in cancer [11–13]. Moreover, p53 has a key role in suppressing Manganese Superoxide Dismutase (MnSOD) expression in response to heat stress-induced oxidative stress in endothelial cells [14]. Similarly to the DNA damage response (DDR) caused by genotoxic stress, a rise of temperature at the cellular level leads to the activation of the ATM kinase DDR pathway that in turn activates p53 [15]. High temperatures inhibit the upstream double-stranded DNA damage sensing machinery mediated by the Mre11-Rad50-Nbs1 (MRN) complex [16]. This suggests alternative regulatory mechanisms leading to the activation of ATM and subsequently to the activation of p53. As p53 is vital for cell homeostasis, variants in *TP53* are specifically associated with environmental adaptation [17, 18].

Concerning the DNA binding domain (DBD) that mediates the transactivation activity of p53, different species have evolved different variations of the p53-DBD complex. Adaptive mechanisms implement variations selected in response to natural living conditions. For example, taxa like the mole-rat *Spalax* and the Salamander *Axolotl* have adapted their p53-DBS in hypoxic environments with certain substitutions identical to human tumour-associated mutations that inhibit hypoxia-induced apoptosis in favour of cell cycle arrest [19, 20]. In addition, geographical diversity can promote evolutionary divergence in metabolism, such as the p53(D228E) variation found in Mediterranean pine voles (*Microtus duodecimcostatus*), but not in Lusitanian pine voles (*M. lusitanicus*) with individuals from the northern region carrying an extended tandem repeat sequence in intron 6 [21].

Temperature affects protein turnover, with the example of p53 demonstrating inherent instability of the human p53(DBD) at temperatures above physiological temperatures (∼44 °C), with a half-life of just ∼9 min at physiological temperature. In addition, temperature-induced DNA-binding characteristics affect the thermodynamic stability of the DBD and lower the unfolding temperature of the DBD in several destabilised and inactivated oncogenic mutants on p53(DBD) [22]. In general, the temperature-dependent effect of such mutations can be direct by preventing the sequence-specific transactivation activity. Or they are indirect, by inducing a local defected structural variant on the DBD that causes the loss of DNA binding activity. For example, the p53(R248A) mutation has a half-life time of 128 min at low temperature compared to less than 3 min at 36.85 °C (310 K), meaning that R248A is more stable at low temperatures than at physiological temperatures [23]. The p53(R248Q) mutation induces both a contact and a structural mutation that binds specifically to several p53 Response Elements (REs) at sub-physiological temperatures 24.85–32.85 °C (298–306 K) but not at 36.85 °C [23]. Interestingly, the wt p53 adopts the R248Q mutant structure at low temperature [23], showing that such mutations can be employed as models to simulate stress-induced conformations of the WT p53. In another example, the V143A mutant aggregates at physiological temperatures, yet it binds DNA at sub-physiological temperatures [22]. Out of the several key residues that also constitute DBD mutations modulating p53’s DNA-binding activity at different temperatures, 40% is concentrated in aa R175, G245, R248, R249, R273 and R282 [23]. This thermodynamic effect on protein kinetics affects the temporal pattern of p53 accumulation, similarly to diverse types of genotoxic stress [24].

In fact, DNA damage and temperature fluctuation are two stressors that seem to be interlinked, influencing both duration and frequency of p53 pulses. Indeed, in the range of 33 °C to 39 °C, pulsatile p53 dynamics are affected [3], modulating the duration of the p53 expression. For example, an exposure of untreated cells to lower temperature induced an increased duration and a reduced frequency of p53 pulses, compared to damaged cells at 37 °C. However, the reverse effect was observed at 39 °C (pulse duration decreased resulting to increased frequency of the pulses) [3]. At temperatures simulating hyperthermia (>40 °C), a hyper-accumulation of p53 was expressed, regardless of the presence of genotoxic stress [3]. Similar thermoregulated dynamics are shown for NF-kB-mediated responses [25].

Intense heat stress is known to lead to oxidative stress and induces mitochondrial translocation of p53 and p53-mediated apoptosis [26]. P53 also promotes survival at 40 °C by preventing a hyper-reactive heat shock response [27]. HSPs modulate the binding of p53 DBD to the promotor of the downstream target CDKN1A/p21 by stabilising the p53-DNA complexes [28]. The Hsp70 and Hsp90 chaperones regulate the conformation of the p53 DBD and hotspot mutants, balancing its conformational plasticity and stability, thus protecting it from unfolding [29]. And structural experimental studies reveal the effect of heat-shock temperatures on the chaperone activity of Hsp90 and specifically on the structural interaction of p53-DBD with Hsp90 [30]. Since p53-DNA binding is required for p53-dependent transcription and p53-mediated activity [23], these observations suggest that hyperthermia could constitute an important factor for improved cancer therapy [3]. Indeed, rescue strategies of mutant-loss of binding at physiological temperature are not unlikely [23]. Furthermore, HSPs implicate post-translational modifications, regulating the interaction of p53 with its main regulator, the MDM2 E3 ubiquitin ligase. For example, HSPs pharmaceutical inhibitors targeting mutant p53-HSP complexes that lead to accumulation of mutant p53 by inactivating the p53 ubiquitination by MDM2, are investigated in clinical trials of non-small cell lung cancer (NSCLC) [31].

In conclusion, there is strong evidence linking p53 activity with temperature fluctuations revealing an underlying interplay between temperature and DNA damage response mediated via cell cycle regulation involving p53 activity. As it happens, elephants might provide answers to key questions concerning underlying mechanisms controlling the response of p53 to fluctuations in the temperature of tissues, cells and cellular components. Why elephants? The short answer is that they have evolved in South Africa from an isolated ancestor pool and have, uniquely for all animals, evolved a multiple set of the TP53 genes that control the assembly of p53. This elevates the elephant to the status of a natural ‘experimental paradigm’, which allows detailed studies of the multiple actions of p53 by probing the action of p53 variants that have evolved naturally in response to powerful selection pressures—rather than having been artificially genetically engineered. While there are ongoing discussions concerning the origin, evolution and selection forces that led to African elephants (*Loxodonta africana*) having 20 copies of TP53 [32, 33]. Notwithstanding, it seems that elephants generically can be temperature challenged because of their dimensions. Importantly, heat stress affects the elephant’s non-descending testicles, which means that their spermatogonia operate at body temperature [34], which for a mammal is a highly unusual situation, with grave implications for sperm production [35]. Hence there is a strong argument for case of the elephant having evolved its p53 complexes primarily to guard the germline genome with the protection of the soma-line being of secondary importance. However, this may be, the multiple copies of p53 allow us to explore the function of this key protein not only in elephants but by extension also in other organisms. We note that other long-living species of large body size, like the Cetaceans (whales), have similarly evolved a high copy number of transcription factors linked to cancer, as for example *CXCR2, ADAMTS8, ANXA1* and *NOTCH3* [36]. Interestingly, regarding whale p53, even though an increased copy number has not been documented [32], a unique leucine substitution in the proline-rich region corresponding to aa 77 in human p53, was exclusively detected in the bowhead whale (*Balaena mysticetus*), who has about four times longer lifespan compared with other whales [37].

In the following, we examine the effect of temperature on the interaction of the multiple copies of elephant p53 with its main regulator, the MDM2 E3 ubiquitin ligase.

## Elephant p53 putative proteins may avoid negative regulation by MDM2 during hyperthermia

The p53 tumour suppressor plays a key role in cell cycle regulation via stress responses in development and ageing as well as in oncogenesis. Under normal conditions, the activation of p53 is negatively regulated by the MDM2 E3 ubiquitin ligase, which binds the BOX-I motif (*FxxxWxxL*) at the N’ terminal of p53, signalling its degradation via the 26S proteasomal pathway. However, in response to cell stress, such as DNA damage, the ATM kinase is activated to prevent the p53-MDM2 protein–protein interaction (PPI) and to activate p53. The elephant’s genome contains several copies of p53 of yet poorly studied functions [32, 33, 38–40]. Studies showed that elephant p53(BOX-I) putative peptides differentially interact with the main regulator of p53, the MDM2 [33]. Two models have been proposed regarding the functionality of these sequences, aiming to address the mechanism whereby these p53 copies contribute to an increased p53 activity in the elephant. The ‘*guardian*’ model suggests that the p53 copies form complexes with the canonical p53 protein, preventing its degradation via MDM2 [32]. The ‘*decoy*’ model suggests that the copies act as decoys for the MDM2 complex allowing the canonical TP53 protein to escape negative regulation [32].

A third model was suggested more recently by Padariya et al. [33]. Supporting that model is the observation that truncated p53 putative proteins (PPs) exhibit a spectrum of reduced binding activity to MDM2. This would provide a range of specialised copy-specific and/or contributing activities that dynamically regulate the activation of p53 in response to various types and amplitudes of stresses [33]. Experiments tested the potential range of the elephant p53 structures (i.e., p53 peptides) and their interaction capacities with MDM2, under normal conditions [33]. This showed that, compared to the canonical elephant p53, the modified BOX-I structures of the p53 PPs exhibit a range of decreased interaction with MDM2. Indeed, the influence of stress conditions, such as genotoxic stress and/or temperature on the structures and the interactions, is anticipated in order to address the dynamic contribution of the p53 PPIs in the p53-MDM2 axis.

**Fig. 1:**
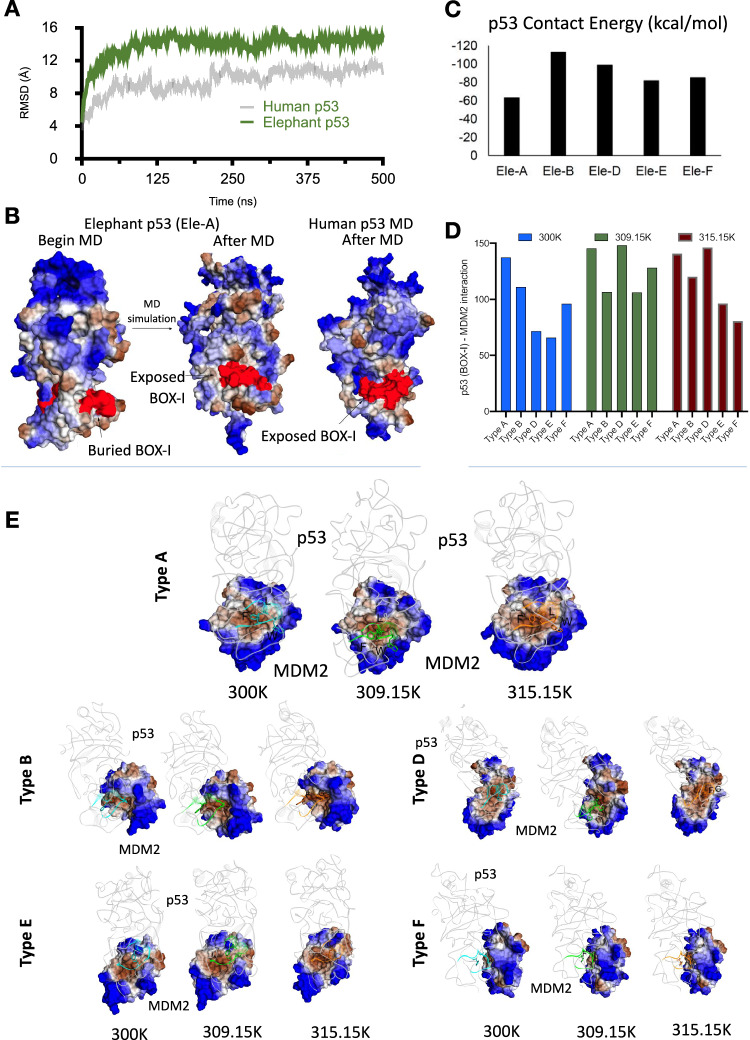
Thermostability, contact energy and Docking Models of p53 proteins interacting with MDM2. **A** RMSD calculation comparing human p53 with canonical elephant p53 shows that the elephant p53 protein adopts a more thermostable conformation. **B** Models comparing the p53 structure before and after MD simulation. The BOX-I motif is more exposed in the elephant compared to human. **C** Contact energy calculation of elephant p53 LoxoPPs including each Type of BOX-I (**A**–**F**). **D** Docking capacity of full-length putative proteins encoding each Type of p53 BOX-I with MDM2 at different temperatures (normal environmental temperature, 300 K (corresponding to 26.85 °C); normal body temperature, 309.15 K (corresponding to 36 °C); and hyperthermia, 315.15 K (corresponding to 42 °C)). **E** Docking models of each interaction calculated in “(**D**)”.

Here we employed a previously experimentally validated in silico approach [33] to test the effect of temperature as a stress factor that potentially regulates the binding of MDM2 to the p53 putative proteins encoded by the *Loxodonta africana* elephant p53 copies. A Root Mean Square Deviation (RMSD) calculation allows for the comparison of the contact energy and thermostability of the p53 protein in human and in elephant. Overall, the elephant p53 protein (NCBI: XP_010594888.1) adopts a more stable conformation compared to human and the BOX-I motif is less exposed to MDM2 (Fig. 1A). The BOX-I motif adopts a buried conformation in elephant compared to a more exposed one in human p53 (Fig. 1B). In addition, the contact energies of MDM2 with each one of the six p53 putative BOX-I structures are different, indicating that the thermostability of the p53 putative proteins is variable (Fig. 1C). Docking models of all six types of BOX-I [33] with MDM2 (NCBI: XP_023414401) (Fig. 1B) tested for the effect of temperature (normal environmental temperature, 26.85 °C (300 K); normal body temperature, 36 °C (309.15 K) and hyperthermia, 42 °C (315.15 K), on the conformation of *Loxodonta africana* elephant putative p53 proteins (LoxoPPs) that include the BOX-I motifs. The type-A (Uniprot: G3T035; canonical elephant p53 X1) peptide, includes the three highly conserved crucial residues of the MDM2-binding motif *FxxxWxxL*, while the type-B (Uniprot: G3UJ00; RTG 17, identical BOX-I to RTG2, 5, 6, 7, 8, 9, 11 and 13), the type-D (Uniprot: G3UI57; RTG4), the type-E (Uniprot: G3ULT4; RTG10) and the type-F (Uniprot: G3U6D1; RTG 16, identical BOX-I to RTG12, 14, 15 and 18) display variations altering the binding affinity to MDM2, as described previously [33].

Testing the effect of temperature on the interaction of these putative proteins with MDM2 by docking models with calculated GBVI/WSA dG (–kcal/ mol) values, demonstrated that these LoxoPPs exhibit a variable temperature-dependent stereochemistry and diverse docking association to MDM2 (Fig. 1D, E). In line with previous findings, all the LoxoPPs have a lower binding affinity to MDM2, as compared to the canonical p53 [33]. At hypothermia Type A (canonical) exhibits an increased docking activity to MDM2 compared to the other p53 sequences (Fig. 1D, E, in blue), which is maintained in normal body temperature (Fig. 1D, E, in green). However, compared to normal body temperature, during hyperthermia only the Type A and Type D are maintained at similarly low levels of kcal/mol, while the rest of the LoxoPPs show variable docking to MDM2. Type B shows a slight decrease (lower kcal/mol value) so it exhibits a better docking during hyperthermia compared to normal body temperature. Types E and F increase (higher kcal/mol), indicating that hyperthermia leads to less good docking in hyperthermia compared to normal body temperature. These results indicate that during hyperthermia, the LoxoPPs encoded by the retrogene copies corresponding to the Types E and F and F of BOX-I, escape the interaction and negative regulation by MDM2, suggesting potential functions independently of the canonical p53 [33]. In addition, these models suggest a competition between the canonical p53 (BOX-I) and Type D BOX-I. Together, these results show that minimal variations on the BOX-I motif of p53, as naturally evolved in the elephant p53 copies, have a functional temperature-dependent effect on the interaction with MDM2. This finding is in line with previous hypotheses that this system involves distinct pools of p53 proteins with variations on the epitopes interacting with MDM2 thus retaining a dynamic capacity to get activated following stress signals [33].

## Conclusions

We expanded studies exploring the metabolic action of p53 linked to heat stress concerning elephants and the assumption that this system has evolved to cope with heat. An experimentally validated in silico model provides evidence for temperature-dependent activation of p53 copies demonstrating that the dynamic interaction of MDM2 with p53 putative proteins has a significant temperature component. The elephant p53 thus provides a valuable model system (paradigm) to study key functions of the p53 transcription factor, a master regulator of cell homeostasis, a state of balance that will be challenged by the impending heating of our climate. In elephants heat stress appears to affect the structural variability of the LoxoPPs, thus disturbing their stability both biochemically (concerning half-life) and at the cellular signalling level concerning stress-dependant interaction of p53 pools with MDM2. Future studies will have to explore and demonstrate whether, and how much, the elephant paradigm may be of relevance for human medicine, but it emerges clearly that elephants provide novel windows to understanding more fully the multiple roles and functions of p53 concerning the protection of cell DNA in both the soma-line and the germ-line.
